# Supplementary material for: Decrease of Pneumococcal Community-Acquired Pneumonia Hospitalization and Associated Complications in Children after the Implementation of the 13-Valent Pneumococcal Conjugate Vaccine (PCV13) in Taiwan
Source: Vaccines (Basel). 2021 Sep 18;9(9):1043. doi: 10.3390/vaccines9091043 (PMC8471531; doi:10.3390/vaccines9091043)
Supplement: Supplementary file 1 [file vaccines-09-01043-s001.zip › vaccines-1340282-SI.pdf]

Supplementary Table S1. Operational definitions.

Invasive procedures	NHI[1] coding
Thoracocentesis	29012B
Exploratory thoracotomy	67002B
Chest intubation	56010B
Thoracoscopic Decortication of Pleura	67047B
Computed tomography-guided aspiration	33103B
Echo-guided aspiration	19007B
**Respiratory failure procedures**	**NHI coding**
Tracheal stent intubation	67046C
Ventilator use	57001B
Non-invasive positive pressure support	57023B
Oxygen therapy	57004C
**Complications of pneumonia**	**ICD-9[2]/ICD-10[3] coding**
pleural effusion	511.9/J90
Empyema	510.9/J86.9
necrotizing pneumonia	513.0/J85.0
pneumatocele	518.89/J98.4
lung abscess	513.0/J85.1
respiratory failure	518.81/J96.9
bacteremia or sepsis	038.2/ R78.81, B95.3, A40.3
hemolytic uremic syndrome	283.11/D59.3

[1] NHI: National Health Insurance;

[2] ICD-9: International Classification of Diseases, 9th revision;

[3] ICD-10: International Classification of Diseases, 10th revision

Supplementary Table S2. The annual incidence[1] of pneumococcal and unspecific community acquired pneumonia hospitalization rate by age during 2005 to 2015.

	**2005**	**2006**	**2007**	**2008**	**2009**	**2010**	**2011**	**2012**	**2013**	**2014**	**2015**
**P-CAP**											
**< 1 year**	79.5	101.5	102.9	88.5	100.5	144.8	116.6	136.8	153.5	126.9	110.8
**1 year**	101.9	154.8	173.7	160.2	164.8	254.0	198.4	204.5	232.0	178.9	181.1
**2~5 years**	109.3	134.2	142.8	157.6	154.1	232.9	194.7	222.7	171.1	138.3	141.4
**6–18 years**	30.1	19.0	14.6	20.9	25.2	28.2	28.9	27.7	23.3	19.7	22.6
**19–65 years**	3.2	2.6	3.0	3.5	3.1	3.6	3.2	3.1	3.3	2.9	2.8
**66 years or older**	33.0	32.0	35.3	37.9	30.6	34.0	31.5	32.0	33.2	27.6	23.6
**U-CAP**											
**< 1 year**	1421.4	1130.8	1010.6	879.9	731.0	916.2	800.6	696.4	663.5	677.3	693.1
**1 year**	1734.2	1424.5	1356.8	1273.9	1065.2	1348.8	1222.5	1182.6	963.9	992.9	1030.5
**2~5 years**	1149.2	919.3	877.9	890.6	827.5	1113.1	1024.7	865.5	669.1	791.4	848.1
**6–18 years**	290.0	195.5	141.5	165.0	201.4	231.0	251.2	217.6	151.2	180.0	224.2
**19–65 years**	212.5	182.5	189.2	205.4	201.8	250.7	258.2	245.6	241.2	234.5	247.9
**66 years or older**	3172.0	3020.4	3258.3	3396.1	3290.0	3863.2	3901.8	3804.3	3823.8	3659.7	3350.2

P-CAP: pneumococcal community-acquired pneumonia; U-CAP: unspecific community-acquired pneumonia

[1]Data are presented as admissions per 100,000 children-year

Supplementary Table S3. Estimated difference of hospitalization incidence rate between pre- (2005-2012) and post-vaccination period (2013-2016) among patients with pneumococcal or unspecific community acquired pneumonia by age groups. (with seasonality adjustment)

	P-CAP				U-CAP			
Age groups	Estimated difference of hospitalization rate[1]	95 % CI	p value	% of change	Estimated difference of hospitalization rate	95 % CI	p value	% of change
< 1 year	-20.60	-30.8 to -10.4	<0.01	44%	38.55	-29.83 to 112.87	0.26	15%
1 year	-18.50	-36.64 to -0.37	0.05	28%	83.93	-0.66 to 181.43	0.05	24%
2~5 years	-17.14	-26.77 to -7.52	<0.01	37%	53.44	-13.24 to 128.35	0.12	21%
6–18 years	-1.53	-4.28 to 1.22	0.28	22%	26.76	0.78 to 56.85	0.05	50%
19–65 years	-0.26	-0.44 to -0.08	<0.01	28%	-0.54	-13.74 to 12.59	0.93	1%
66 years or older	-2.99	-4.36 to -1.62	<0.01	35%	-148.23	-289.18 to -30.08	0.02	15%

P-CAP: pneumococcal community-acquired pneumonia; U-CAP: unspecific community-acquired pneumonia

[1] hospitalization rate: admission episodes per 100,000 person-years
